# Disulfide Bonds Reduce the Toxicity of the Amyloid Fibrils Formed by an Extracellular Protein**

**DOI:** 10.1002/anie.201100986

**Published:** 2011-06-10

**Authors:** Maria F Mossuto, Benedetta Bolognesi, Bernat Guixer, Anne Dhulesia, Federico Agostini, Janet R Kumita, Gian G Tartaglia, Mireille Dumoulin, Christopher M Dobson, Xavier Salvatella

**Affiliations:** Institute for Research in Biomedicine (IRB Barcelona)Baldiri Reixac 10, 08028 Barcelona (Spain), Fax: (+34) 934-037-114; Joint BSC–IRB Research Programme in Computational BiologyIRB, Barcelona (Spain); Department of Chemistry, University of Cambridge (UK)UPF, Barcelona (Spain); Centre for Genomic Regulation (CRG)UPF, Barcelona (Spain); Centre for Protein Engineering, University of Liège (Belgium)Barcelona (Spain); Institució Catalana de Recerca i Estudis Avançats (ICREA)Barcelona (Spain)

**Keywords:** amyloid fibrils, disulfide bonds, protein aggregation, protein folding, protein stability

The misfolding of proteins into amyloid fibrils constitutes the hallmark of many diseases.[1] Although relatively few physicochemical properties of protein sequences—charge, hydrophobicity, patterns of polar and nonpolar residues, and tendency to form secondary structures—are sufficient to rationalize in general terms their relative propensities to form amyloid fibrils,[2, 3] other properties can also be important. One example is intramolecular disulfide bonds, which limit the ways in which a polypeptide can be arranged in a fibril through the topological restraints that they impose. Although disulfide bonds are present in 15 % of the human proteome, in 65 % of secreted proteins, and in more than 50 % of those involved in amyloidosis, our understanding of how they influence the properties of amyloid fibrils is limited.[4–6] We have examined the formation of fibrils by human lysozyme[7, 8] in the presence and absence (Figure 1 a,b) of its native disulfide bonds, and found that they profoundly influence the fibrillar morphology and cytotoxicity.

**Figure 1:**
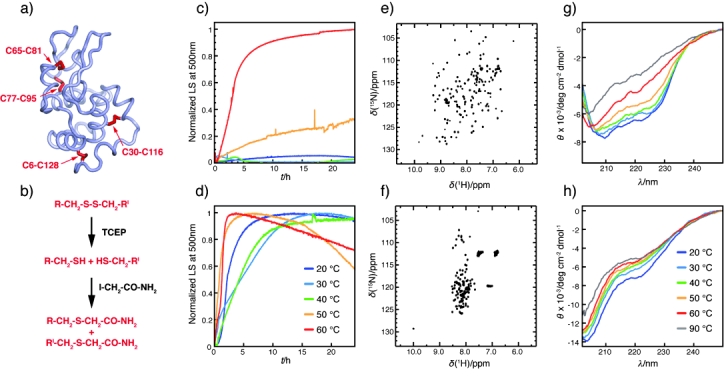
a) Structure (pdb code 1Lz1) of wild-type lysozyme (Lys) with the disulfide bonds shown in red. These were reduced as shown in (b) to obtain Lys^RA^. c, d) Amyloid formation by Lys (c) and Lys^RA^ (d) monitored by light scattering (LS) at 500 nm and different temperatures. e, f) ^1^H–^15^N NMR HSQC spectra show that e) Lys is folded whereas f) Lys^RA^ is disordered. g, h) Effect of temperature on the far-UV CD spectrum of Lys (g) and Lys^RA^ (h). TCEP=tris(2-carboxyethyl)phosphine hydrochloride.

As disulfide bonds stabilize folded proteins, they determine the conditions under which wild-type (Lys) and reduced and alkylated lysozyme (Lys^RA^) are amyloidogenic. In agreement with previous reports, we found that it is necessary to incubate Lys under destabilizing conditions, such as low pH (pH 2.0) and high temperature (≥50 °C), to form amyloid fibrils within 24 h (Figure 1 c and Figure S1 in the Supporting Information).[8–10] By contrast, Lys^RA^ is amyloidogenic under milder conditions; at pH 2.0, for example, it forms fibrils at 20 °C (Figure 1 d and Figure S1 in the Supporting Information). We analyzed the conformational properties of Lys and Lys^RA^ by NMR spectroscopy and far-UV circular dichroism (CD) as a function of temperature. We found that Lys is folded at 20 °C (Figure 1 e,g) and experiences a well-defined unfolding transition at about 55 °C (Figure 1 g and Figure S2 in the Supporting Information).[10] By contrast, Lys^RA^ is unfolded at all temperatures (Figure 1 f,h). Our results, therefore, indicate that the presence of intact disulfide bonds decreases the rate at which lysozyme forms fibrils (Figure 1 c,d) by stabilizing the cooperatively folded native protein.[11]

Disulfide bonds also determine the morphology of the fibrils. After 24 h of incubation under the mildest conditions that lead to aggregation (Figure 1 c,d), both Lys and Lys^RA^ had converted into fibrils as shown by transmission electron microscopy (TEM; Figure 2 a,b insets) and by thioflavin T (ThT) and Congo red (CR) binding (Figure 2 c,d and e,f, respectively). We analyzed the samples by far-UV CD and found that in both cases the spectra evolved from those corresponding to largely disordered proteins (Figure 2 a,b, blue) to those of species rich in β-sheet structure, with a minimum in the ellipticity at approximately 217 nm typical of amyloid fibrils (Figure 2 a,b, red). We also analyzed the amide I region (1580–1720 cm^−1^) of the infrared spectra of the fibrils by using attenuated total reflectance Fourier-transform infrared (ATR-FTIR) spectroscopy (Figure 3 a,b and Table S1 in the Supporting Information),[9] and found that Lys^RA^ fibrils are less rich in β-sheet structure than those formed by Lys (51.5 versus 72.5 %).

**Figure 2:**
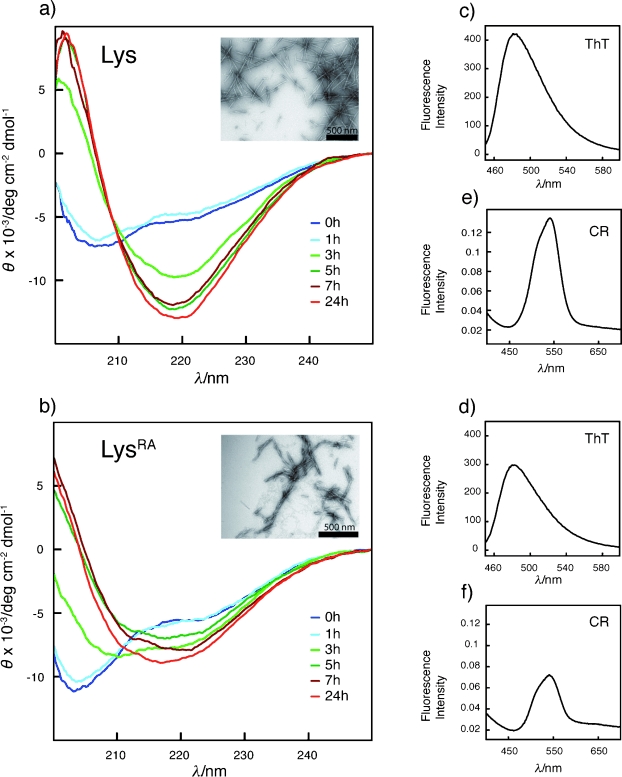
Aggregation kinetics of a) Lys at pH 2.0 and 60 °C and b) Lys^RA^ at pH 2 and 20 °C monitored by far UV-CD. Samples of fibrils formed after 24 h and isolated by ultracentrifugation have a fibrillar morphology (insets in (a) and (b)) and bind ThT (c,d) and CR (e,f). In the presence of the fibrils the ThT fluorescence maximum is ca. 485 nm, and the CR absorbance maximum is ca. 540 nm, which is typical of the amyloid structure (see the Supporting Information).

**Figure 3:**
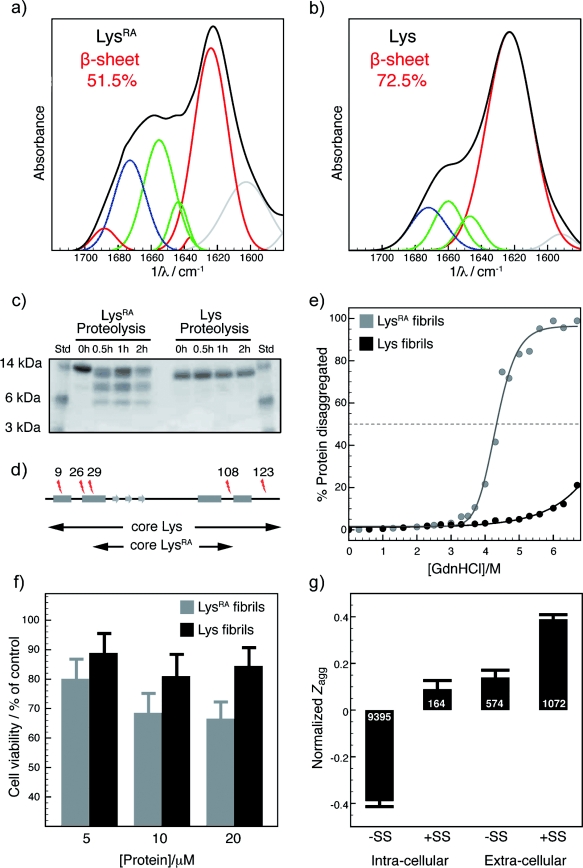
a, b) ATR-FTIR spectra of Lys^RA^ (a) and Lys fibrils (b), shown in black, with the contributions obtained by curve fitting colored as follows: red: β sheet, green: random/α helix, blue: turns and loops, gray: side chains. c) SDS-PAGE of fibril samples isolated by ultracentrifugation during proteolysis. d) Positions of cleavage sites indicated on the polypeptide chain; rectangles and arrows indicate the location of the native α helices and β strands. The protease-resistant region of Lys^RA^ fibrils encompasses residues 29–108; almost exactly the same region (32–108) was found to be protease-resistant in early aggregates of Lys,[24] but maturation progressively transforms these species into fibrils protected from proteolysis (E. Frare, personal communication). e) Disaggregation by GdnHCl of fibrils formed by Lys^RA^ (gray) and Lys (black). f) Effect of fibrils formed by Lys^RA^ (gray) and Lys (black) on SH-SY5Y cells evaluated by the calcein viability assay; the mean and the 95 % confidence interval after three experiments are reported. g) Normalized aggregation propensity of the human proteome as a function of cellular localization and of the presence of disulfide bonds. The numbers in white represent the number of sequences belonging to each class.

To analyze the nature of the fibrillar core of the fibrils, we studied both types of fibrils by limited proteolysis (Figure 3 c,d). It was found that the fibrils formed by Lys are inert to proteolysis under the conditions used here but that those formed by Lys^RA^ are readily cleaved (Figure 3 c,d and Figure S3 in the Supporting Information) and have their diameter reduced from (5.1±0.6) to (3.6±0.6) nm (Figure 2 b and Figure S4 in the Supporting Information). The protease-resistant segment of the molecule, attributable to the core, is composed of about 80 residues, from residue 29 to 108. As susceptibility to proteases requires 10 to 12 unfolded residues,[12] our result is consistent with the number of residues in the β-sheet secondary structure determined by FTIR analysis (51.5 %, that is, 67 residues). We also probed the nature of the non-core regions by an 8-anilinonaphthalene-1-sulfonate (ANS) binding assay, in which interactions of this dye with solvent-exposed hydrophobic patches cause a blue shift in the maximum emission wavelength and an increase in emission intensity.[13] We found that the fluorescence intensity of ANS is higher in the presence of Lys^RA^ fibrils than in that of fibrils formed from Lys (Figure S5 in the Supporting Information), hence indicating a greater number of solvent-exposed hydrophobic residues.

Since hydrogen-bonding interactions in the cross-β core stabilize amyloid fibrils,[14] we investigated whether differences in core size are reflected in their resistance to disaggregation. We measured the concentration of protein in equilibrium with fibrils at increasing concentrations of guanidine hydrochloride (GdnHCl)[15] and found that the fibrils formed by Lys^RA^ disaggregate at lower concentrations of GdnHCl than those formed by Lys (Figure 3 e). Our results indicate that the fibrillar core formed in the presence of disulfide bonds is larger than in their absence, thereby reducing the susceptibility of the fibrils to proteolysis and increasing their stability.

Current evidence suggests that the most toxic forms of amyloid aggregates are not the mature fibrils but their less organized precursors.[16] In addition, recent studies have shown that partially structured fibrils can also give rise to toxicity as a result of their larger accessible hydrophobic area or by their greater tendency to generate toxic oligomeric species by fragmentation.[9] To investigate whether or not disulfide bonds alter the cytotoxicity of the fibrils, samples corresponding to protein concentrations of 5 to 20 μm were added to cultures of SH-SY5Y human neuroblastoma cells and the resulting changes in cell viability were measured using a calcein acetoxymethyl (AM) assay (Figure 3 f). The results, supported by an MTT assay (MTT=3-(4,5-dimethylthiazol-2-yl)-2,5-diphenyltetrazolium bromide; Figure S6 in the Supporting Information), show that the fibrils formed by Lys^RA^ have a significantly higher cytotoxic effect than those formed by Lys (*p*<0.0001; Figure 3 f), in agreement with the finding that ANS binding in amyloid species (Figure S5 in the Supporting Information) correlates with cytotoxicity.[17] This result shows that disulfide bonds can decrease toxicity by favoring the formation of highly structured amyloid fibrils, which suggests that disulfide bonds in extracellular proteins could be the result of evolutionary pressures[18] to minimize toxic aggregation in an environment where the redox potential favors disulfide bond formation.

To investigate this hypothesis further, we analyzed the aggregation propensity of the human proteome using the well-established Zyggregator predictor.[3] We found that the sequences of extracellular proteins have higher intrinsic aggregation propensity than intracellular ones, an observation that has been related to the dilution that occurs upon secretion.[19, 20] We also found that disulfide bonds are associated with sequences of high aggregation propensity (Figure 3 g), which suggests that disulfide bonds have co-evolved with protein sequences[21] to minimize their propensity to form potentially toxic amyloid aggregates. This analysis would explain the high prevalence of disulfide bonds in extracellular proteins, where additional protective mechanisms that reduce misfolding and its consequences are likely to play a less significant role than inside the cell.[20, 22]

The disulfide bonds of lysozyme inhibit the aggregation of this protein into amyloid fibrils by stabilizing the folded state—a fact that can be attributed to the reduction in the entropy of the unfolded state.[11] A partially unfolded state can nevertheless be populated as a result of changes in conditions, as in this work, or by mutations, as in patients with nonneuropathic systemic amyloidosis.[8] We have shown that, when this situation occurs, disulfide bonds allow the formation of fibrils with a large proportion of their sequence in the cross-β conformation. This result is at first sight unexpected, as one might have anticipated that the conformational constraints resulting from cross-linking would reduce the ability of the chain to fold into the complex β-sheet amyloid structure. It is, however, clear that lysozyme and other disulfide-linked proteins are able to form fibrils that contain a high fraction of sequence in the cross-β structure.[23]

We conclude that intramolecular disulfide bonds can stabilize amyloid fibrils, as they do for the folded state, by decreasing the entropic penalty associated with the formation of this ordered form of protein structure. This can be concomitant with significant decreases in the toxicity of the resulting fibrils, which suggests that disulfide bonds have co-evolved with protein sequences to reduce toxic aggregation.[21]

## Experimental Section

The disulfide bonds of lysozyme were reduced with tris(2-carboxyethyl)phosphine hydrochloride and the free thiol groups protected with iodoacetamide to prevent disulfide bond formation; control experiments (Figure S7 in the Supporting Information) showed that protection does not alter the biophysical properties of the reduced protein. The ^1^H–^15^N NMR HSQC spectra shown in Figure 1 e and f were recorded at pH 2.0 and 20 °C. Aliquots of the aggregation mixture were withdrawn and diluted with the aggregation buffer to give a final protein concentration of 7 μm immediately prior to recording the spectra shown in Figure 2 a and b. The species obtained after proteolysis of the Lys^RA^ fibrils were isolated, disaggregated, and analyzed by mass spectrometry to identify the protein fragments. Differences in cell viability were assessed using a Wilcoxon test and aggregation propensities were calculated using the Zyggregator algorithm.[3]
