# Supplementary material for: Disulfide Bonds Reduce the Toxicity of the Amyloid Fibrils Formed by an Extracellular Protein
Source: Angew Chem Int Ed Engl. 2011 Jun 10;50(31):7048–51. doi: 10.1002/anie.201100986 (PMC3166663; doi:10.1002/anie.201100986)
Supplement: Supplementary file 1 [file anie0050-7048-SD1.pdf]

Angewandte Chemie
Eine Zeitschrift der Gesellschaft Deutscher Chemiker

Supporting Information

© Wiley-VCH 2011

69451 Weinheim, Germany

Disulfide Bonds Reduce the Toxicity of the Amyloid Fibrils Formed by an Extracellular Protein**

*Maria F. Mossuto, Benedetta Bolognesi, Bernat Guixer, Anne Dhulesia, Federico Agostini, Janet R. Kumita, Gian G. Tartaglia, Mireille Dumoulin, Christopher M. Dobson, and Xavier Salvatella**

anie_201100986_sm_miscellaneous_information.pdf

Supplementary Text

Material and methods

Materials

Human lysozyme, 1-anilino-naphthalene-8-sulfonic acid (ANS) and Thioflavin-T (ThT) were purchased from the Sigma-Aldrich (St. Louis, MO). All other chemicals were of analytical reagent grade and were obtained from Sigma-Aldrich (St. Louis, MO).

Methods

Reduction and alkylation of human lysozyme - Wild-type human lysozyme (Lys) was dissolved at 0.1mM in the reduction buffer (6M GdnHCl, 0.1M TrisHCl, pH 8.5), a 10-fold molar excess of tris(2-carboxyethyl) phosphine hydrochloride was then added and the pH adjusted to 8.5. After incubation at 25°C for 2 hours, a 10-fold molar excess (over the total number of sulfhydryl groups in the protein) of iodoacetamide was added. The mixture was incubated 1 h at 25°C in the dark and then dialyzed extensively against 1% formic acid (pH 2.0). The purity of the reduced and alkylated protein (Lys^{RA}) was checked by SDS-PAGE and RP-HPLC. Quantitative derivatization of sulfhydryl groups was verified by ESI mass spectrometry. An increase in mass of 464.2 Da, relative to the Lys, was observed, corresponding to the addition of a carbamidomethyl group to each one of the eight cysteine residues of the lysozyme molecule.

Optical spectroscopy - Protein concentrations were evaluated from absorption measurements at 280 nm on a single-beam Cary 400 Scan spectrophotometer (Varian, Palo Alto, CA, USA). The extinction coefficients at 280 nm, calculated with the method of Gill & von Hippel (1989) [1], were 36940 cm^{-1} M^{-1} for Lys and 36440 cm^{-1} M^{-1} for Lys^{RA}.

Fluorescence measurements were carried out on a Varian (Palo Alto, CA, USA) model Cary Eclipse spectrofluorimeter in a temperature-controlled cell holder, utilizing a 2 mm x 10 mm path length cuvette. ThT binding was monitored by exciting the sample at 440 nm and recording the emission fluorescence spectrum from 450 to 600 nm. For each measurement, 8 µl of a 2.5 mM ThT stock solution prepared in PBS were added to a volume of fibrils corresponding to 20 µg and a volume of 0.5 ml was reached with the phosphate buffer. For the Congo red spectroscopic assay, 50 µl of a 70 µM fibril sample was added to 1 ml of 5mM phosphate buffer, 150mM NaCl pH 7.4 containing 1mM Congo red. The UV spectra between 400 and 700 nm were immediately acquired at 20°C: the difference between the resulting spectra and the one measured with only Congo red in the absence of protein was used as the effective bound Congo red absorbance [2,3]. For 8-anilo-1-naphtalene-sulfonic acid (ANS) titration, aliquots of ANS from a stock solution in 20 mM glycine (pH 2.0) containing 0.1 M NaCl, were added to the isolated fibrils, to a final ANS concentration ranging from 0 to 200 µM. The final protein concentration was 5 µM in all cases. The spectra were immediately acquired at 20°C, using an excitation wavelength of 350 nm and an emission range from 380 to 700 nm. The difference between the resulting fluorescence intensity at 470 nm and that measured with only ANS in the absence of protein was used as the effective bound ANS fluorescence. The extinction coefficient at 350 nm of ANS was 4950 cm^{-1} M^{-1} [4].

Far-UV CD spectra of Lys and Lys^{RA} were acquired in 20 mM glycine·HCl, 100 mM NaCl pH 2.0. Proteins were diluted to a final concentration of 7 μM and the spectra were acquired at 20 or 60°C using a 1 mm pathlength cuvette and a J-810 Jasco spectropolarimeter (Tokyo, Japan), equipped with a thermostated cell holder.
For secondary structure analysis of fibrils, samples were analyzed in a Bruker BioATRCell II using a Bruker Equinox 55 Fourier transform infrared spectroscopy (FTIR) spectrometer (Bruker Optics Limited, UK) equipped with a liquid nitrogen cooled mercury cadmium telluride (MCT) detector and a silicon internal reflection element (IRE). For each spectrum 256 interferograms were coadded at 2 cm^{-1} resolution, and the buffer background was independently measured and subtracted from each protein spectrum before curve fitting of the amide I region (1720-1580 cm^{-1}). Calculation of the second derivatives was used to identify peak maxima. Using this information, the raw spectra were then fitted to a series of Gaussian peaks with the identified absorbance maxima using an iterative curve-fitting procedure performed in Origin8 (OriginLab Corporation, MA, USA).

NMR spectroscopy of Lys and Lys^{RA} at pH 2.0 - Uniformly ^{15}N-labelled human lysozyme was expressed and purified as described previously[5], by growing cells with $^{15}(NH_4)_2SO_4$ as the sole nitrogen source. NMR samples containing 70 μM ^{15}N-labelled Lys or Lys^{RA} lysozyme in 0.1 M NaCl, 20 mM glycine buffer pH 2.0, made with 95% (v/v) H_2O/5% (v/v) ^2H_2O were prepared. 2D ^{15}N–^1H HSQC spectra were measured at 293 K in a Bruker Digital Avance Spectrometer at 800 MHz.

SDS-PAGE analysis - Fibrillar samples, previously dissolved in DMSO, were analyzed by SDS-PAGE using 4%-12% Bis-Tris NuPAGE gels (Invitrogen UK) in MES buffer under reducing conditions. Gels were stained using coomassie brilliant blue.

RP-HPLC and Mass Spectrometry - RP-HPLC analyses were performed on a Vydac C18 column (4.6 mm × 150 mm; The Separations Group, Hesperia, CA) eluted with a linear gradient of acetonitrile containing 0.05% (v/v) trifluoroacetic acid from 5% to 22% in 5 min and from 22% to 50% in 17 min, at a flow-rate of 0.6 ml/min. The effluent from the column was monitored by measurement of the absorbance at 226 nm. The identity of protein fragments was assessed by nanoelectrospray mass spectrometry (NanoESI-MS) with a LTQ-FT Ultra mass spectrometer (Thermo Scientific, USA). The monoisotopic mass values were analyzed using massXpert.

Transmission Electron Microscopy - Samples were applied to formvar-coated nickle grids, stained with 2% (w/v) uranyl acetate solution and imaged on a JEOL 1010 transmission electron microscope operating at 80kV. Images were taken with a Megaview III camera and digitized with the software AnalySIS (Soft Imaging System). TEM images were analysed using ImageJ.

Aggregation monitored by right-angle light-scattering - Aggregation studies were performed with Lys and Lys^{RA} lysozyme samples (70 μM, 0.1 M NaCl, 20mM Glycine buffer pH 2.0), incubated with stirring at various temperatures (20-60°C) in a Cary Eclipse spectrofluorimeter (Varian Ltd., Oxford UK). Light-scattering was monitored at 500 nm with slit-widths of 5 nm.

Formation of Lys^{RA} and Lys amyloid fibrils at pH 2.0 - Lys and Lys^{RA} fibrils were prepared by dissolving the proteins in a 0.1 M NaCl, 20 mM glycine buffer pH 2.0 at a concentration of 70 µM and stirring the solution at 500 rpm for 24 h. Samples of fibrils were characterized by ThT and Congo Red binding and TEM and the endpoint samples from the aggregation reactions were ultracentrifuged (90000 rpm, 4°C, 45 min). The insoluble material was dissolved in DMSO and analyzed by SDS-PAGE, reverse phase high performance liquid chromatography (RP-HPLC) and mass spectrometry to confirm that degradation of lysozyme had not occurred.

Measurement of conformational stability - Aliquots of fibrils were diluted into buffered solutions containing increasing concentrations (0-7 M) of GdnHCl and after 72 h at 20°C, the samples were ultracentrifuged at (90000 rpm, 20°C, 45 min). The concentration of lysozyme in the supernatant was evaluated by measuring the absorbance at 280nm. The disaggregation curves were obtained by plotting the fraction of lysozyme released from the fibrils at given concentrations of denaturant.

Proteolysis of fibrils - Lysozyme fibrils, isolated by ultracentrifugation and resuspended at 70 µM, were subjected to proteolysis in the aggregation buffer at 20°C, using pepsin at an enzyme:substrate ratio of 1:50 (w/w). After proteolysis, fibrils isolated by ultracentrifugation were dissolved in DMSO for SDS-PAGE analysis and in 7.4 M GdnHCl under shaking for analysis by RP-HPLC.

Cell Culture - SH-SY5Y cells were cultured in Dulbecco's Modified Eagle Medium (Invitrogen) with the addition of 10% fetal bovine serum at 37°C in a humidified 5% CO_2 incubator. The cells were plated in Costar (3595) 96-well plates (Corning) using serum-free Neurobasal medium (GIBCO) and were then incubated with 1 µM aggregates for 48 h. The percentage of viable cells present after 48 h was assessed by adding Calcein AM and measuring fluorescence of calcein, the product of its hydrolization by intracellular esterases, and by adding MTT and measuring the absorbance of the product of its reduction by mitochondrial dehydrogenases.

Proteome analysis and aggregation propensity – The set of all entries belonging to Human Complete Proteome was downloaded from Uniprot (2010_12, [6], www.uniprot.org) and redudancy was eliminated at a sequence identity threshold of 100%. All entries with a localization associated with the membrane, the ones marked as fragments and the proteins with an existence level termed "uncertain" were fuiltered out from the dataset. The set of proteins was divided in 4 classes: intracellular with disulfide bonds, intracellular without disulfide bonds, extracellular with disulfide bonds, extracellular without disulfide bonds. Classification of every entry was done on the basis of the Uniprot annotation (presence or absence of disulfide bonds and annotated cellular location).

For the calculation of the intrinsic propensity profiles for aggregation of polypeptide sequences, the intrinsic aggregation propensities of individual amino acids are defined as:

$$p_i^{agg} = \alpha_h p_h + \alpha_s p_s + \alpha_{hyd} p_{hyd} + \alpha_c p_c \qquad (1)$$

where p_h and p_s are the propensities for α helix and β sheet formation of the amino acid at position i and p_{hyd} and p_c the hydrophobicity and charge, respectively. The p_i^{agg}

values are combined to provide a profile, A^p, which describes the intrinsic propensity for aggregation as a function of the complete amino acid sequence (1). At each position i along the sequence we define the profile A^p as an average over a window of seven residues:

$$A_i^p = \frac{1}{7}\sum_{j=-3}^{3} p_{i+j}^{agg} + \alpha_{pat} I_i^{pat} + \alpha_{gk} I_i^{gk} \quad (2)$$

where I_i^{pat} is the term that takes into account the presence of specific patterns of alternating hydrophobic and hydrophilic residues and I_i^{gk} is the term that takes into account the gatekeeping effect of individual charges c_i:

$$I_i^{gk} = \sum_{j=-10}^{10} c_{i+j} \quad (3)$$

In order to compare the intrinsic propensity profiles of different proteins, we normalized A^p by considering the average (m_A) and the standard deviation (s_A) of A_i^p at each position i. We thus obtain the normalized intrinsic aggregation propensity profile:

$$Z_i^p = \frac{A_i^p - \mu}{\sigma} \quad (4)$$

We calculated the average μ and the standard deviation σ over a set of random sequences:

$$\mu = \frac{1}{(N-8)\cdot N_S}\sum_{k=1}^{N_S}\sum_{i=4}^{N-4} A_i^p(S_k) \qquad \sigma^2 = \frac{1}{(N-8)\cdot N_S}\sum_{k=1}^{N_S}\sum_{i=4}^{N-4}\left(A_i^p(S_k) - \mu\right)^2 \quad (5)$$

The parameters α are determined by minimizing the error between positive values of Z_i^p (i.e., the Zagg score) and experimental aggregation rates.

In the present study, the Zagg score is normalized using Eq. (4) and 20400 human sequences available from Uniprot (http://www.uniprot.org/).

In the analysis of intracellular and extracellular sequences, proteins with sequence similarity higher than 50% were filtered out. For this purpose, the CD-HIT algorithm (http://weizhong-lab.ucsd.edu/cd-hit/) was applied to the four classes (i.e., intracellular with disulfide bonds, intracellular without disulfide bonds, extracellular with disulfide bonds, extracellular without disulfide bonds) that were employed for the analysis. The number of proteins employed in this work is reported below:

Secreted proteins	without disulfide bonds:	574
Secreted proteins	with disulfide bonds:	1072
Non-secreted proteins	without disulfide bonds:	9395
Non-secreted proteins	with disulfide bonds:	164

With respect to the statistical significance of Zagg the un-equal variance t-test was used: i) Extracellular proteins with and without disulfide bonds are discriminated by Zagg with p<=0.001, ii) Intracellular proteins with and without disulfide bonds are discriminated by Zagg with p<=0.001.

Supplementary Figures

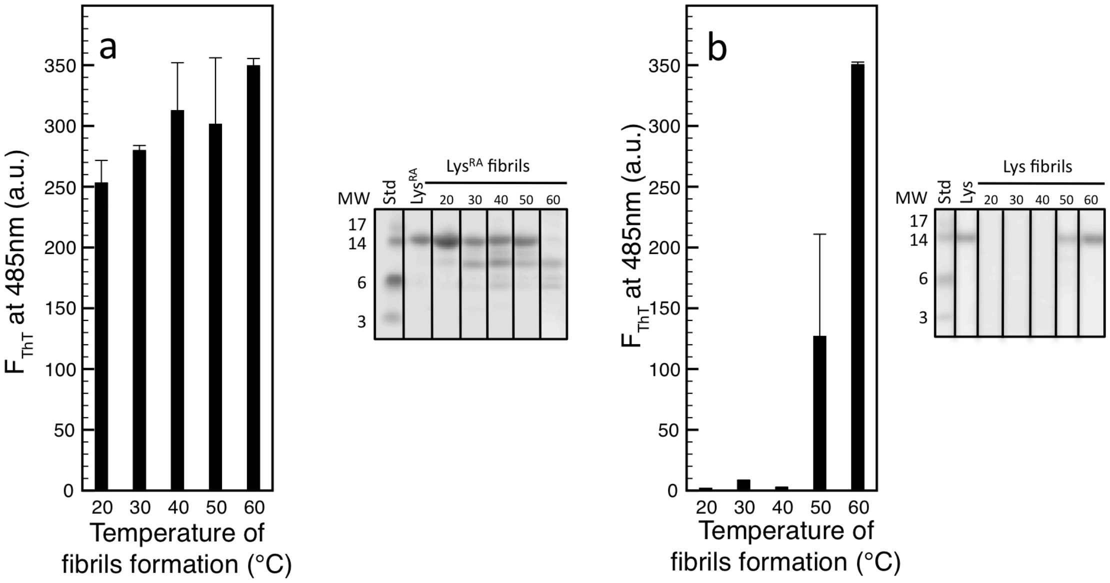

Fig. S1 - Thioflavin T fluorescence in the presence of Lys^{RA} (a) and Lys (b) samples after 24 h of aggregation at different temperatures (see Figure 1 c-d). SDS-PAGE of Lys^{RA} (a) and Lys (b) fibrils isolated by ultracentrifugation after 24h of aggregation at different temperatures. As the aggregation temperature increases Lys^{RA} undergoes more acid-catalyzed hydrolysis of Asp-X peptide bonds [7] and the fibrils are formed by a larger amount of protein fragments. However, for the Lys protein, the presence of fragments in the fibrils is negligible. The different sensitivity of the two proteins to acid-catalyzed hydrolysis of Asp-X peptide bonds is likely due to the fact that the Lys^{RA} protein is more unfolded and therefore more exposed and flexible than the Lys protein.

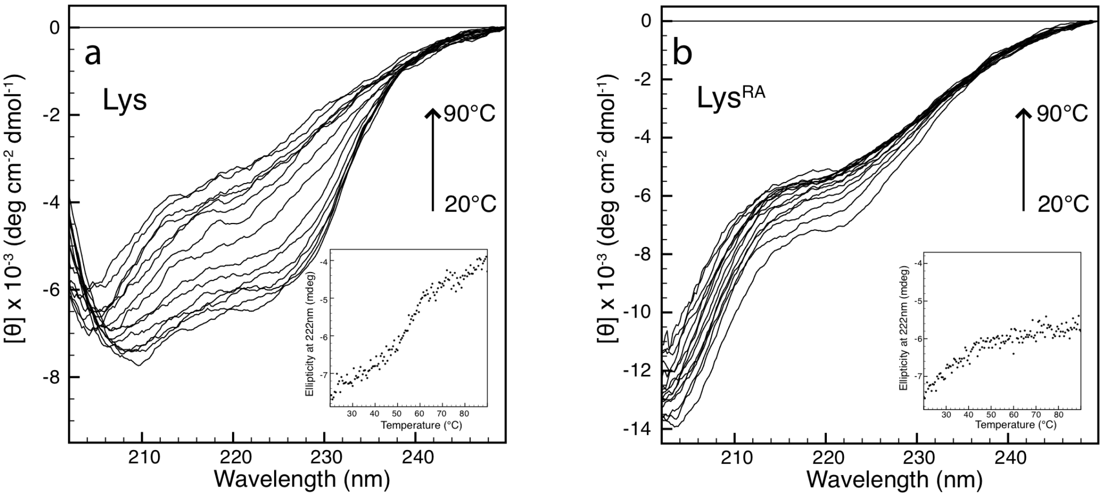

Fig. S2 - The effect of temperature on the secondary structure of Lys (a) and Lys^{RA} (b) was studied by far-UV CD spectroscopy. The insets describe the change in the CD signal at 222 nm, reporting on the α-helical structure present in the sample, during the thermal unfolding experiments.

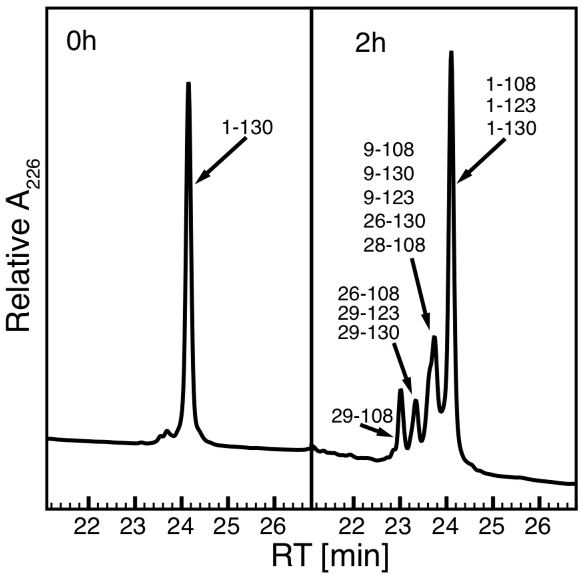

Fig. S3 - RP-HPLC chromatograms of the Lys^{RA} fibrils isolated by ultracentrifugation after 0 h and 2 h of proteolysis. The fibrils obtained by ultracentrifugation after proteolysis were dissolved in 7.4 M GdnHCl and analyzed by RP-HPLC. The identity of the various protein species was determined by MS and is given by the labels near the chromatographic peaks.

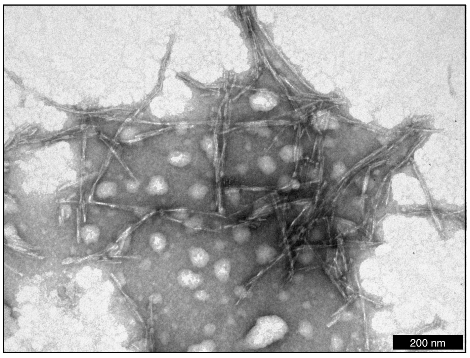

Fig. S4 - TEM image of Lys^{RA} fibrils after proteolysis.

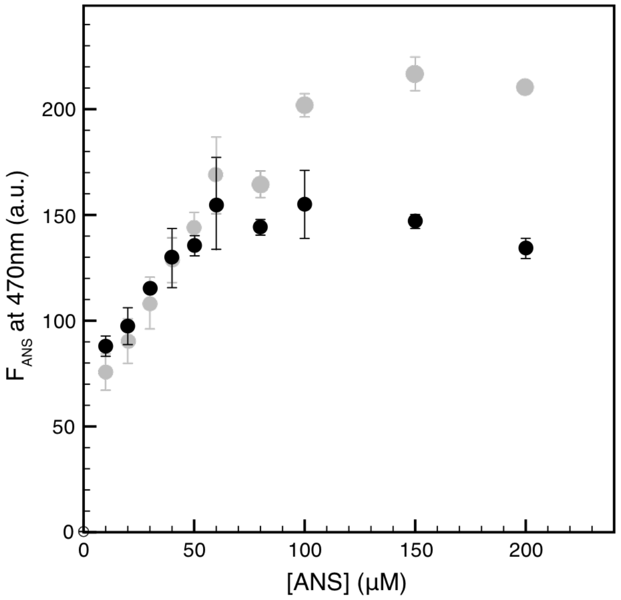

Fig. S5 - ANS binding to Lys^{RA} (grey) and Lys (black) fibrils. The ANS fluorescence intensity measured at 470 nm is reported as a function of the ANS concentration. The protein concentration was 5 μM in all cases.

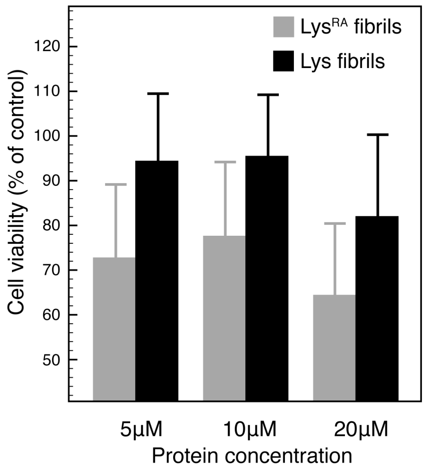

Fig. S6 - Effect of the addition of Lys^{RA} (grey) and Lys (black) fibrils on SH-SY5Y neuroblastoma cells tested by the cell viability MTT assay. SH-SY5Y cell viability was assessed in the presence of increasing concentrations (from 5 to 20 µM) of Lys^{RA} and Lys fibrils. The mean and the 95% confidence interval after three experiments are reported ($p = 0.0082$).

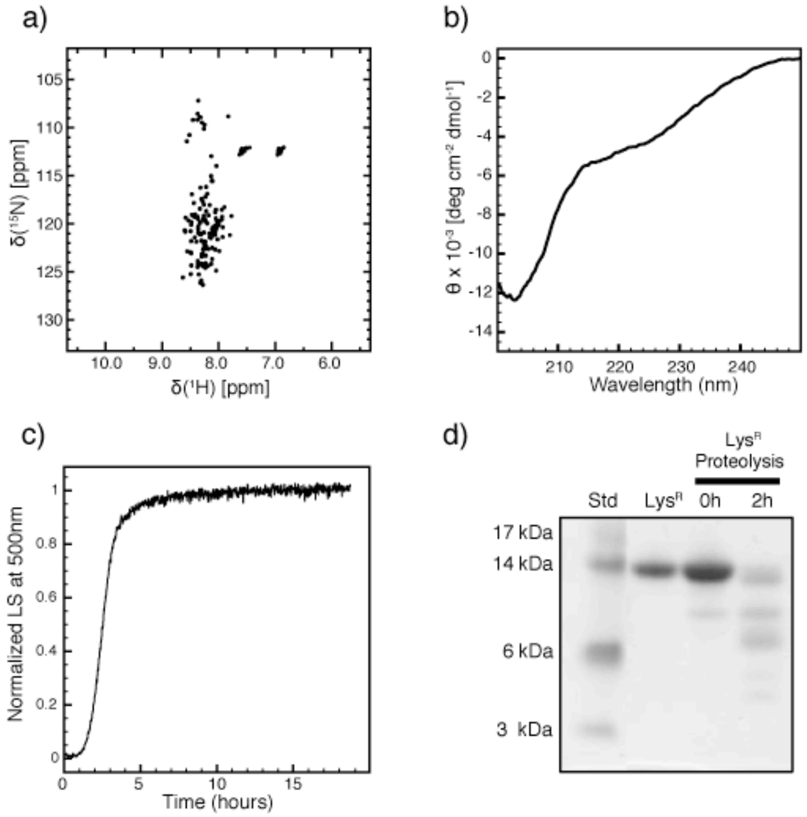

Fig. S7 – (a) ^{1}H-^{15}N HSQC spectrum of reduced but not alkylated lysozyme (Lys^{R}) (b) far-UV CD specrum of Lys^{R} (c) kinetics of fibril formtion by Lys^{R} monitored by light scattering at 500 nm and (d) SDS-PAGE of samples of fibrils formed by Lys^{R} isolated by ultracentrifugation during proteolysis. All experiments were carried in the presence of 2 mM tris(2-carboxyethyl) phosphine hydrochloride.

Supplementary Tables

Table S1 - Secondary structure content of fibrils formed by Lys^{RA} and Lys as determined by curve fitting of the ATR-FTIR spectra shown in Fig. 3.

	Lys^{RA} fibrils		Lys fibrils	
**Assignment** [a]	**cm^{-1}** [b]	**%** [c]	**cm^{-1}** [a]	**%** [b]
β-sheet	1624	46.9	1623	72.5
	1636	1.0		
Random/ α-helix	1644	5.9	1647	6.2
	1655	23.1	1660	10.1
Turns/loops	1673	19.5	1672	11.2
β-sheet	1688	3.6		

[a] Classification of the bands obtained from curve-fitting of FTIR spectra: bands centered between 1620-1640 cm^{-1} and between 1680-1700 cm^{-1} were assigned to β-structure, bands between 1640-1660 cm^{-1} to random structure and α-helices and bands between 1660-1680 cm^{-1} to turns or loops.
[b] Peak position of the amide I band components, as deduced by the second derivative spectra.
[c] Percentage area of the amide I band components, as obtained by integrating the area under each deconvoluted band. The areas corresponding to side chain contributions located at 1580-1610 cm^{-1} were not considered in the deconvolution.

Table S2 - Analytical characterization of the peptide fragments obtained by the proteolysis of Lys^{RA} fibrils (see Fig. 3)

Retention Time[a]	Observed Mass (Da)[b]	Calculated Mass (Da)[c]	Identity of the fragment
23.1	9044.3	9044.1	29-108
23.5	9415.5	9415.3	26-108
	11070.3	11070.1	29-123
	11834.7	11834.4	29-130
	5958.8	5958.7	51-103
23.8	11304.6	11303.3	9-108
	14094.9	14093.7	9-130
	13331.6	13330.4	9-123
	12205.8	12205.6	26-130
	9230.4	9230.2	28-108
	5744.8	5746.0	51-101
24.2	12367.1	12365.9	1-108
	14393.1	14391.8	1-123
	15157.5	15156.2	1-130

[a] Retention times corresponding to the HPLC fractions shown in the chromatograms of Fig. S3
[b] Determined by ESI-MS
[c] Molecular masses calculated from the amino-acid sequence

Supplementary References

[1] S. C. Gill, P. H. von Hippel, *Anal. Biochem.* **1989**, *182*, 319-326.
[2] W. E. Klunk, J. W. Pettegrew, D. J. Abraham, *J. Histochem. Cytochem.* **1989**, *37*, 1293-1297.
[3] M. R. Nilsson, *Methods* **2004**, *34*, 151-160.
[4] M. Cardamone, N. K. Puri, *Biochem J.* **1992**, *282*, 589-593.
[5] J. R. Kumita, R. J. Johnson, M. J. Alcocer, M. Dumoulin, F. Holmqvist, M. G. McCammon, C. V. Robinson, D. B. Archer, C. M. Dobson, *FEBS J.* **2006**, *273*, 711-720.
[6] *Nucleic Acids. Res.* **2010**, *38*, D142-8.
[7] A. Li, R. C. Sowder, L. E. Henderson, S. P. Moore, D. J. Garfinkel, R. J. Fisher, *Anal. Chem.* **2001**, *73*, 5395-5402.
